# Aerodynamic Analysis of Rotor Spacing and Attitude Transition in Tilt-Powered Coaxial Rotor UAV

**DOI:** 10.3390/s24227115

**Published:** 2024-11-05

**Authors:** Wei Wu, Xinyu Tan, Xing Liu, Angang Luo, Lanjie Niu

**Affiliations:** 1 School of Electronic Information Engineering, Xi’an Technological University, Xi’an 710021, China; tanxinyu@st.xatu.edu.cn (X.T.); lxing@xatu.edu.cn (X.L.); 2 School of Mechatronic Engineering, Xi’an Technological University, Xi’an 710021, China; luoangang@st.xatu.edu.cn; 3 Science and Technology on Electromechanical Dynamic Control Laboratory, Xi’an 710065, China; nljwl@163.com

**Keywords:** coaxial rotor, rotor layout, numerical analysis, parameter optimization, CFD

## Abstract

Complex aerodynamic characteristics and optimal control during the attitude transition of tilt-powered coaxial twin-rotor unmanned aerial vehicles (UAVs) represent key challenges in flight control design. This study investigates aerodynamic mechanisms and control parameter optimization during the transition of UAVs from vertical to forward flight. By establishing a dynamic model and combining theoretical and numerical analyses, the optimal rotor spacing is determined to be h = 0.5 R. The load distribution and aerodynamic characteristics of the aircraft are analyzed at different initial tilt angles during attitude transitions. At an initial tilt angle of δ = 9°, the thrust force increases by 439% compared with that at δ = 3°, and the tip speed increases by 15% and 35% compared with that at δ = 3° and δ = 13°, respectively. The results indicate that a tilt angle of δ = 9° results in a higher turbulent dissipation rate and rotor layout efficiency, with a smoother vortex flow and more orderly distribution. The interference between the twin-rotor tip vortices is relatively weak, resulting in excellent symmetry and aerodynamic stability. Through the improvement of the theoretical model and parameter optimization of a novel tilt-powered coaxial twin-rotor UAV, this study enhances UAV flight stability and provides valuable insights and validation for the further development of UAV technology.

## 1. Introduction

In recent years, unmanned aerial vehicles (UAVs) have played a critical role in both military and civilian applications, demonstrating broad development prospects. With the continuous advancement of the UAV industry, coaxial twin-rotor UAVs have gradually come into focus [1,2,3,4,5]. These UAVs are designed with a simple structure, offering capabilities for short-range vertical takeoff and landing (VTOL) and hovering. Their foldable wings increase their portability, making them well suited for deployment by individual soldiers. Additionally, they are equipped to carry out precision strike missions, such as ground assaults or escort operations, which underscores their value for military research and applications.

A coaxial twin-rotor UAV is structurally designed with two coaxial rotors, where the torque produced by the two rotors counterbalances each other under the condition of maintaining a constant heading. This stability in heading is essential for the torque to effectively neutralize each other, allowing the UAV to generate lift and control its flight direction. You et al. studied the kinematic and dynamic characteristics of foldable coaxial twin-rotor UAVs, providing a reference for the optimization of flight control parameters and the development of control strategies [6]. Hayami et al. applied high-precision computational fluid dynamics (CFD) to analyze the aerodynamic performance of isolated coaxial rotors under forward flight conditions [7]. In the subsequent sections of this paper, theoretical and numerical analyses are combined to establish a corresponding mathematical model, and CFD methods are employed to analyze the aerodynamic performance of UAVs.

Despite the advantages of coaxial twin-rotor UAVs, such as small size, portability, and VTOL capabilities, they are limited by slower flight speeds and shorter endurance. In comparison, tilt-rotor UAVs combine the benefits of both fixed-wing and multirotor UAVs, offering higher speeds and longer endurance. Due to these advantages in flight speed, endurance, and maneuverability, researchers have conducted extensive studies on flight characteristics, control methods, and aerodynamic behavior during the mode transitions of tilt-rotor UAVs to address challenges in design and flight control. Lv et al. designed a novel coaxial tilt-rotor UAV and investigated its control methods [8]. Vuruskan et al. developed a mathematical model for the transition flight of a tilt-rotor VTOL UAV. They determined the aerodynamic coefficients for the transition flight state through a CFD analysis and evaluated the UAV’s flight attitude at various speeds at attack angles ranging from 0° to 15° [9]. Öner et al. proposed a new tilt-wing UAV, developed its mathematical model, and presented control algorithms for vertical flight [10]. Li et al. introduced a multi-layer moving embedded grid technique to reduce numerical dissipation in wake flows during mode transitions and conducted a comparative analysis of aerodynamic forces in different flight modes for tilt-rotor UAVs [11].

**Figure 1:**
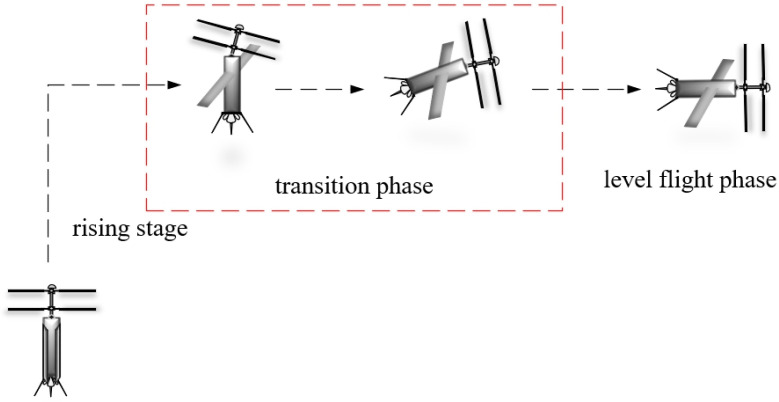
Attitude adjustment process of coaxial twin-rotor UAV.

As research into tilt-rotor UAVs has progressed, it has become evident that traditional designs often combine multirotor and fixed-wing configurations. In contrast, the coaxial twin-rotor structure offers superior stability and efficiency to multirotor designs. Therefore, this study focuses on a novel tilt-powered coaxial twin-rotor UAV, which integrates a coaxial contra-rotating rotor structure with a fixed-wing configuration. By adjusting the tilt angle of the coaxial twin-rotors, this UAV can transition to horizontal flight mode, achieving higher speed and an extended range. The attitude transition process is illustrated in Figure 1.

In the field of aerodynamics, many scholars have conducted extensive research on the aerodynamic characteristics of micro-aerial vehicles through wind tunnel experiments [12,13,14,15]. Wind tunnel testing is a commonly used method in aerodynamic research; however, it is costly, time-consuming, and lacks flexibility. With advances in computer hardware and algorithms, the accuracy and precision of CFD simulations have improved significantly, allowing flexible model and parameter adjustments with lower costs and higher efficiency. Wang et al. used Fluent 2023 R2 software to perform CFD simulations on the blades of foldable coaxial twin-rotor UAVs, employing the sliding mesh method and the k-ε SST turbulence model. The results were consistent with wind tunnel data, with an error margin not exceeding 12.46% [16]. Xin et al. used seagull-like and NACA 4412 airfoil profiles to design straight wings, conducted numerical simulations using Fluent software, and performed wind tunnel tests on scaled-down wings, finding consistency between the simulation and test results [17]. Howell et al. proposed a design and study of the aerodynamic performance of small vertical-axis wind turbines (VAWTs), combining experimental and simulation methods [18]. Taking into account uncertainties in both CFD simulations and wind tunnel measurements, the simulation results matched the experimental data. These studies demonstrate the reliability of CFD simulation methods. Considering cost-effectiveness, flexibility, and controllability, the current study employs CFD simulations to analyze the aerodynamic characteristics of UAVs.

Shukla and Komerath examined the aerodynamic characteristics of rotors hovering at different rotor spacings and Reynolds number ranges, revealing that a smaller rotor spacing and lower Reynolds numbers resulted in stronger wake interactions and reduced rotor efficiency [19]. Lee, H. and Lee, D.J. performed aerodynamic analyses on small multirotor UAVs, studying the effects of rotor tip spacing on aerodynamic and acoustic characteristics, concluding that increasing the rotor spacing reduced the interference between rotors [20]. Lee et al. developed a free-wake model to analyze the aerodynamic characteristics of twin-rotor systems and investigated wake instabilities caused by wake interactions [21]. These studies highlight the significant impact of the rotor configuration on UAV aerodynamics [22]. The present study aims to determine the optimal spacing between the upper and lower rotors and the optimal tilt angle during the transition process, with the goal of improving the aerodynamic stability of UAVs.

Many studies on traditional tilt-rotor UAVs, which integrate multirotor and fixed-wing designs, remain focused on the VTOL and forward flight stages [23,24,25]. Vera and James conducted numerical simulations to investigate the aerodynamic interference between various components of a compound coaxial helicopter in forward flight [26]. Mark et al. performed CFD simulations of tilt-rotor hover configurations [27]. However, there is a lack of aerodynamic analyses concerning the attitude adjustment process during the transition from ascent to forward flight. Due to the complex aerodynamic behavior during mode transitions in tilt-rotor UAVs, this area remains a key focus and challenge in research. Therefore, this research focuses on the aerodynamic characteristics during attitude transitions.

The focus of this study is a novel tilt-powered coaxial twin-rotor UAV, which exhibits complex aerodynamic behavior during its attitude transition process. The mechanisms involved remain unclear. To address this issue, a mathematical model is developed, incorporating a nine-degree-of-freedom dynamic equation to study the relative motion between the rotor blades and the payload beneath during the attitude transition process. This enables a detailed analysis of the forces acting on the coaxial twin-rotor unit and the payload during the transition from ascent to horizontal flight. Using a CFD analysis, the optimal spacing between the upper and lower rotors is determined during the design phase. Additionally, the overall aerodynamic characteristics of different initial tilt angles during the attitude transition are analyzed to determine the optimal initial tilt angle. The ultimate goal is to refine the theoretical model and optimize the parameters of the novel tilt-powered coaxial twin-rotor UAV, improving its flight stability and control performance. Achieving rapid and efficient attitude transitions will significantly advance UAV technology.

## 2. Models and Methods

### 2.1. Coaxial Twin-Rotor UAV Model

**Figure 2:**
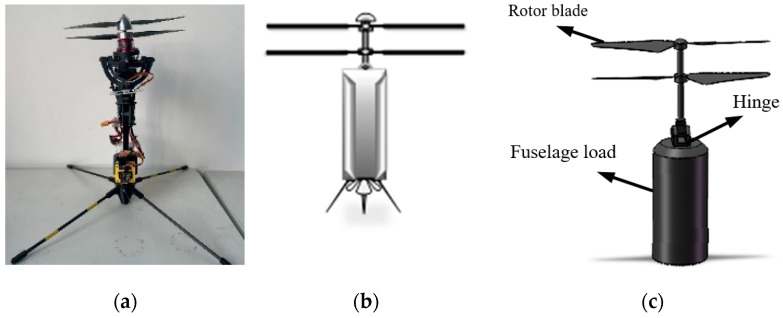
Coaxial twin-rotor UAV model. (**a**) Physical model. (**b**) Theoretical model. (**c**) Simplified model.

A coaxial twin-rotor UAV is structured with a pair of rotors that are coaxial and rotate in opposite directions; the rotational speed of its rotors is controlled by two motors, and the change in the rotor tilt angle is controlled by two servos. Physical and structural conceptual drawings of a coaxial twin-rotor UAV are shown in Figure 2a,b. SolidWorks 2023 software is used here to build the model, and the model is simplified in the final application process due to the need for aerodynamic simulations at a later stage, as well as the fact that the initial tilt angle of the UAV attitude transition process is examined in this study, which does not take into account the expansion of the fixed-wing. The simplified model is shown in Figure 2c.

From its structure, it can be seen that the paddle layout of this coaxial twin-rotor UAV is divided into two parts, upper and lower, with each part controlled by the corresponding motor. The two motors rotate at the same speed in opposite directions to realize the coaxial dual-rotor effect. The vertical takeoff and landing process of the coaxial dual-rotor UAV can be realized by controlling the rotational speed change in the motors. If the level flight process of the coaxial twin-rotor UAV is to be realized, the overall tilt angle of the coaxial twin-rotor structure is controlled by the servo to realize the power control of vertical flight to horizontal flight.

### 2.2. Flow Field Division

A sliding mesh (SM) model is used for the motion of the upper and lower rotor blades of the model, and the total computational domain is defined as a cylinder with a height of 3.7 m and a radius of 1 m. In order to ensure the accuracy of the numerical simulation, the computational region is divided into four flow field domains, namely, the upper rotation domain, the lower rotation domain, the inner flow field, and the outer flow field.

**Figure 3:**
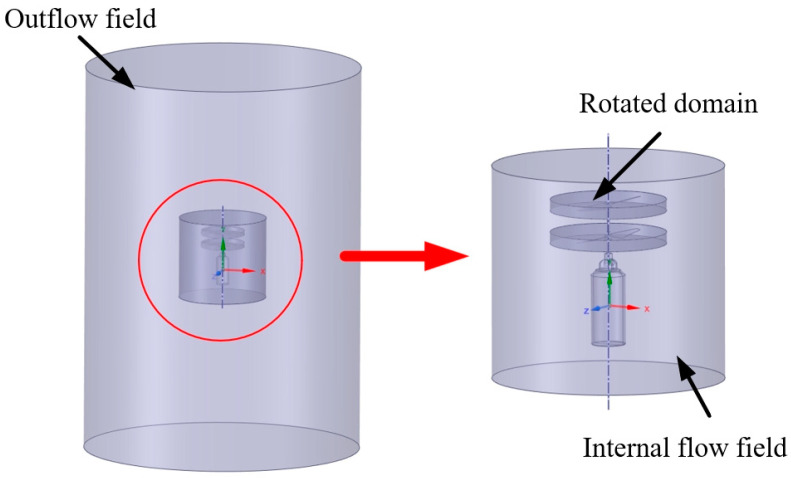
Schematic diagram of flow field division.

The upper and lower rotation domains use the slip grid method to realize the coaxial twin-rotor inversion effect. Because of its grid movement, the subsequent grid division needs to be encrypted in order to ensure the accuracy of the calculation results. The inner flow field is connected to the upper and lower rotational domains, and there is a contact surface between the flow field and the dynamic grid, so the subsequent grid processing should be encrypted appropriately. The outer flow field is only connected to the inner flow field, without considering the grid movement. A schematic diagram of the flow field division is shown in Figure 3.

### 2.3. Meshing

In this study, Fluent software is used to perform an aerodynamic simulation of the coaxial twin-rotor UAV model. The two rotational domains are set to have the same rotational speed and opposite directions, and the rotational domains are used to encrypt the mesh to improve the accuracy of the simulation. The external flow field is divided into two parts in order to improve the computational accuracy and speed; the internal flow field is set to be connected to the rotational domain, which is meshed with more node cells and has better quality, while the external flow field is meshed with fewer node cells in order to improve the computational speed of the simulation. An unstructured grid is used to provide higher simulation precision and accuracy.

A set of 30 prismatic layers is applied around the rotor blades, with a grid growth rate of 1.2. The first element on the geometry wall is set to 0.23 mm in order to ensure that the dimensionless wall distance, $y^{+}$, remains below 1. The dimensionless $y^{+}$ is calculated using the following equation:

(1) $$y^{+}=\frac{\rho yu_{t}}{\mu }$$

**Figure 4:**
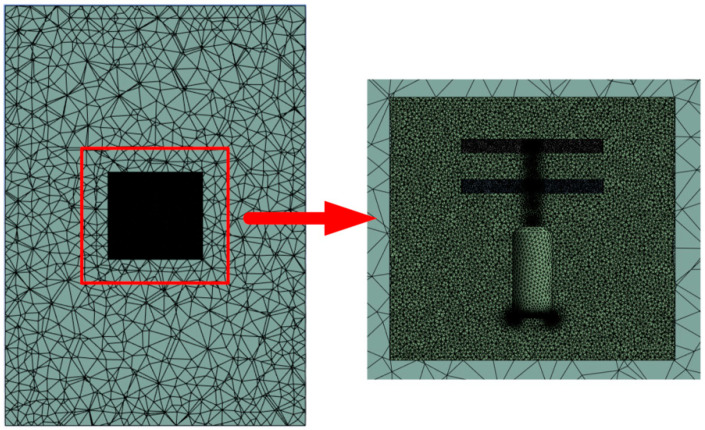
Schematic diagram of grid division.

Here, ρ, y, $u_{t}$, and μ represent the fluid density, normal wall distance, friction velocity, and fluid viscosity, respectively. A schematic of the mesh division is shown in Figure 4.

### 2.4. Calculation Methods and Boundary Condition Settings

In this study, it is known that the highest speed is at the tip of the rotor blade of the UAV, where the highest rotor speed set is 7200 RPM, so the rotor blade tip linear speed can be calculated as follows:

(2) V=ωR=7200/60×2π×0.122≈92m/s

According to the definition of the Mach number, we can obtain the Mach number Ma < 0.3 at the rotor tip, and, as the air in the rotor flow field is an incompressible fluid and its compressibility is negligible, we choose the pressure-based solver.

In this study, the computational domain is a cylinder, and the rotation of the two paddles is simulated by the relative motion between the meshes; additionally, the rotational speed of the rotor is set to 7000 RPM, where the air outflow field is regarded as a low-speed unpressurized fluid. The inlet surface is set as the velocity inlet, and the outlet surface is set as the pressure outlet. The interface between the rotational domain and the stationary domain (computational domain) is set. An implicit time discretization scheme is employed for the time-stepping method, with a total of 140 rotor revolutions. Considering the effect of a low Reynolds number, the RNG k-*ε* turbulence model is selected [28]. A swirl-dominated flow is used to facilitate cyclonic corrections in order to improve solution accuracy, and the standard wall function is selected.

### 2.5. Validation of Numerical Simulation Methods

**Figure 5:**
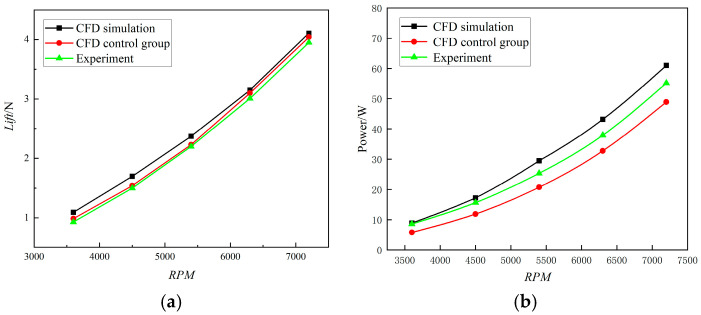
Simulation of DJI Phantom single-rotor at different rotational speeds vs. simulation control and experimental groups. (**a**) Comparison of lift at different speeds. (**b**) Comparison of power at different speeds.

In order to verify the reliability of the turbulence model and solution method used in this study, the data obtained are compared with Yoon’s CFD simulation data [29] and Zawodny’s DJI Phantom experimental data [30]. The lift data of the DJI Phantom UAV single-rotor wing show the trend of its lift with the change in rotational speed, as shown below. Under the RNG k-*ε* turbulence model and the solution method chosen in this study, CFD simulations are performed for the speeds of 3600 RPM, 4500 RPM, 5400 RPM, 6300 RPM, and 7200 RPM, which were used in the previous paper. The trend of the single-rotor lift with rotational speed is compared with Yoon’s CFD simulation data and Zawodny’s DJI Phantom experimental data, as shown in Figure 5.

The calculations based on the speeds of 3600 RPM, 4500 RPM, 5400 RPM, 6300 RPM, and 7200 RPM are compared with the results of the comparison group. The error is greatest when the rotational speed is 3600 RPM, with the error of the simulation control group data and experimental data being 10% and 17%, respectively. As the rotational speed increases, the simulation results obtained in this study better fit the experimental data. The inaccuracy is the lowest when the rotational speed is increased to 7200 RPM; it is only 1.5% and 3.75% for the experimental and simulation control group data, respectively. Therefore, the reliability of the turbulence model and solution method is determined to be good.

Figure 5b shows that the power variation trend in the simulation group closely aligns with the validation group, following a linear growth relationship. This outcome corresponds with the characteristic of electric power loss in the motor, which increases linearly with RPM. The consistency in the power trend is well validated, further demonstrating the reliability of the simulation results. Therefore, it can be concluded that the turbulence model and solution method used in this study are highly reliable.

### 2.6. Grid-Independent Verification

**Figure 6:**
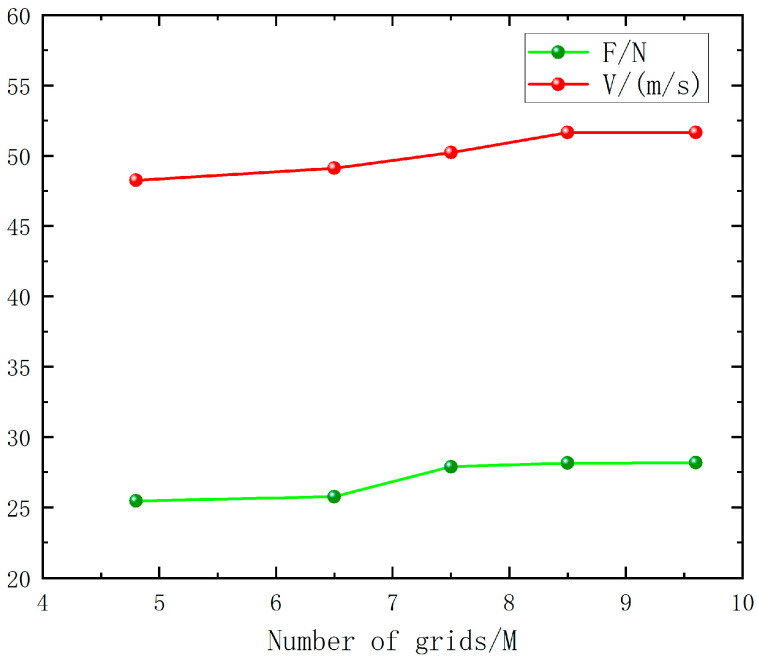
Lift and velocity data at different grid densities.

Considering that the number of grids affects the accuracy of the calculation results, the effects of different grid numbers on the numerical analysis results are systematically compared while keeping the other conditions unchanged. The total number of grid cells of the model increased from 4,883,899 to 9,644,588, and the corresponding results are recorded, as detailed in Figure 6.

This table presents the lift and velocity data of the coaxial twin-rotor UAV model under vertical takeoff and landing conditions with different grid densities. Based on the above data analysis, it can be seen that the calculation results show a stable trend after the number of grid cells exceeds 8,533,928. A thrust convergence verification was conducted through Richardson extrapolation, indicating that the numerical simulation results have high accuracy. Considering the accuracy and speed of the calculation results, it is recommended that the number of grid cells should be higher than 8.5 M for the simulation and that the number of grid cells selected for the subsequent calculations in this study meets the above criteria.

## 3. Results and Discussion

### 3.1. Effect of Rotor Spacing

The total lift $F_{T}$ of the coaxial twin-rotor system is the sum of the upper rotor lift $F_{u}$ and the lower rotor lift $F_{l}$, expressed as follows:

(3) $$F_{T}=F_{u}+F_{l}=C_{T}\rho A\Omega^{2}R^{2}(1+\eta )$$

Here, the interference factor η is a function of the rotor spacing h, representing the aerodynamic interference between the upper and lower rotors. The specific form is as follows:

(4) $$\eta \left(\frac{h}{R}\right)=1-K_{1}e^{-K_{2}\left(\frac{h}{R}\right)}$$

In this equation, the empirical coefficients $K_{1}$ and $K_{2}$ are related to the rotor design and aerodynamic characteristics.

The analysis of the forces on the rotor model above shows that there is a loss of aerodynamic efficiency between the upper and lower rotors during normal operation. The combined force generated by these two rotors is the total lift of the UAV during the takeoff and landing phases, as well as the initial phase of the attitude transition process. Therefore, by studying the effect of different rotor spacings on the lift, the rotor layout can be optimized to improve the aerodynamic performance of the model.

According to the comparison in the previous section, the aerodynamic simulation results are closer to the experimental data when the rotational speed is 7000 RPM, so the rotational speed is taken to be 7000 RPM. To avoid inter-rotor collision, as well as considering the space size of the whole airplane, the spacing range is determined to be between 0.2 R and 0.7 R. The rotor spacing is defined as h, and the spacing ratios h/R are taken as 0.2, 0.3, 0.4, 0.5, and 0.7 to obtain the lift of the coaxial twin-rotor at different spacings.

**Figure 7:**
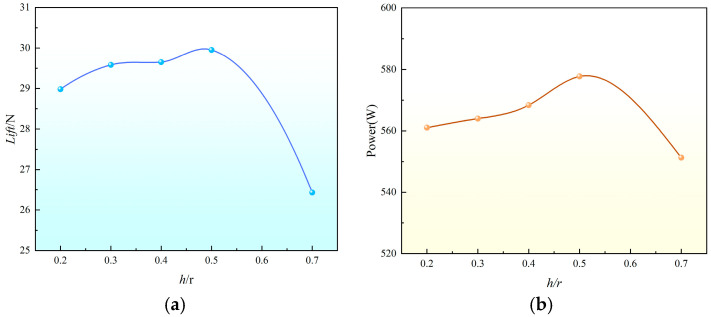
Change in coaxial twin-rotor at different spacings. (**a**) Lift of different spacings. (**b**) Power of different spacings.

In an analysis of Figure 7a, it can be seen that when the spacing ratio h/R is increased from 0.2 to 0.5, the rotor lift shows an increasing trend. When the spacing ratio is greater than 0.5, with the increase in spacing, the upper and lower rotor interaction effects are weakened, the coaxial lift decreases, and the overall performance decreases. At this time, the rotor lift at h/R = 0.5 is 3% higher than that at h/R = 0.2, and that at h/R = 0.5 is 13% higher than that at h/R = 0.7. Therefore, when the rotor pitch ratio is h/R = 0.5, the resulting lift value has obvious advantages. According to Figure 7b, it is clear that the power performance at a spacing of 0.5 outperforms other spacings, further supporting the conclusion that it is the optimal spacing. At this spacing, the rotor layout efficiency is also higher.

**Figure 8:**
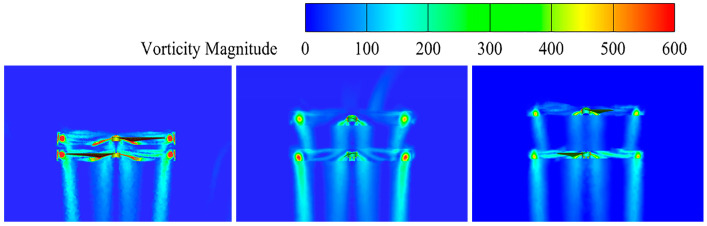
Vorticity of coaxial twin-rotor at different spacings.

Based on the above, this study selects rotor spacings of 0.2 R, 0.5 R, and 0.7 R for simulation verification, and vorticity maps, velocity maps and pressure maps are obtained. From the maps of vorticity under different rotor spacings, the evolution of rotor wake can be observed. In a coaxial twin-rotor system, the wakes of the two rotors interact with each other, and the effects of this interaction vary depending on the rotor spacing. An analysis of Figure 8 shows that when the spacing between the upper and lower rotors is small, the lower rotor is subjected to strong interference from the wake of the upper rotor, leading to a significant vortex effect. As the rotor spacing increases, the wake generated by the upper rotor partially dissipates before reaching the lower rotor, thereby reducing its influence. This reduction in interference results in a weakening of the overall vortex intensity.

**Figure 9:**
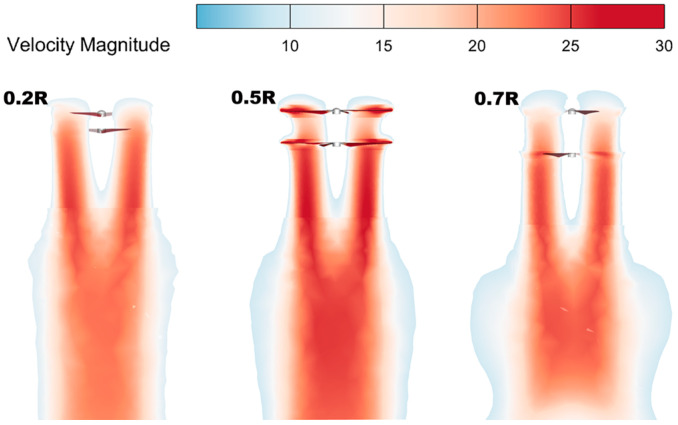
Velocity maps of coaxial twin-rotors with different spacings.

The velocity maps can characterize the velocity size of the blade profiles; the larger the induced velocity of the downwash airflow of the rotor, the weaker the aerodynamic interference between the upper and lower rotors, and the stronger the superposition effect of the downwash airflow under the twin-rotors, the better the aerodynamic performance of the coaxial twin-rotor system [31]. As shown in Figure 9, the induced velocity of the rotor downwash airflow increases when the rotor spacing increases from 0.2 R to 0.5 R. When the spacing increases to 0.7 R, the induced velocity of the rotor downwash tends to decrease.

**Figure 10:**
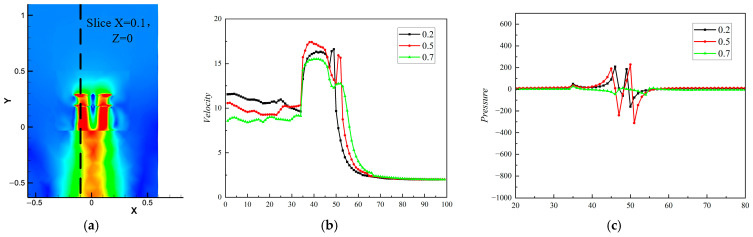
Slicing coaxial twin-rotor velocity and pressure data plots for X = 0.1 and Z = 0. (**a**) Slicing schematic. (**b**) Velocity data. (**c**) Pressure data.

When the spacing is 0.5 R, the induced velocity of the lower rotor downwash airflow is the largest (the area of the red part in the velocity maps is the largest), which is sliced, as shown in a, by selecting X = 0.1 and Z = 0. As can be seen in Figure 10b, when the spacing is 0.5 R, the integrated velocity of the slices is the largest, and the maximum velocity of the slices is improved by 7% and 12% compared with that at spacings of 0.2 R and 0.7 R, respectively; thus, the aerodynamic performance of the coaxial twin-rotor system is better at this time.

The pressure difference between the upper and lower surfaces of the rotor in the pressure maps can be used to characterize the amount of rotor lift. Additionally, the lift of the upper and lower rotor surfaces can be deduced from the change in pressure distribution at spacings of 0.2 R, 0.5 R, and 0.7 R. The pressure data in Figure 10c show that, when the rotor spacing is 0.5 R, the pressure difference at the rotor is obviously larger than that at the other rotor spacings, with a maximum pressure difference of 540 Pa. When the rotor spacing is 0.7 R, the differential pressure at the slicing position is obviously reduced. Analyze these results, it is determined that the reason for this phenomenon is that the pressure difference tends to decrease and shrink to the center when the rotor spacing is too large, and the slices taken in this study are located at the edge of this pressure center.

**Figure 11:**
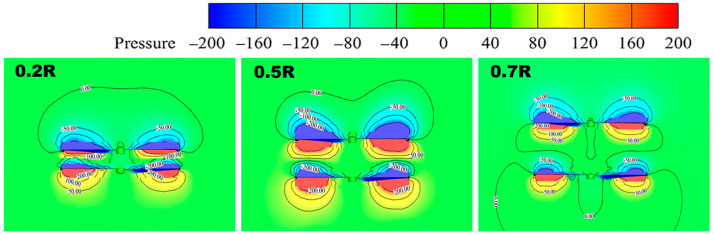
Coaxial twin-rotor pressure maps at different spacings.

In Figure 11, it can be seen that, when the spacing increases to 0.5 R, the difference in pressure between the upper and lower surfaces of the rotor gradually increases, and the lift of the system increases. When the spacing increases to 0.7 R, the pressure difference between the upper and lower surfaces of the rotor becomes smaller, and the corresponding lift decreases. Therefore, according to the pressure maps, when the rotor spacing is 0.5 R, the lift of the coaxial twin-rotor system is large and stable, and the aerodynamic performance is good.

In summary, it can be seen that a spacing too large or too small is not conducive to the stability of aerodynamic performance: when the spacing is too small, the aerodynamic interference is strong, and when it is too large, the downwash airflow effect is weakened, the favorable interference between the rotor blade is reduced, and the overall performance is reduced. Thus, it is very important to select a suitable rotor blade spacing ratio in order to enhance overall aerodynamic performance. By combining the rotor lift, velocity distribution, and pressure distribution changes, the optimal rotor pitch h = 0.5 R is selected, at which time the rotor lift and the overall aerodynamic performance have obvious advantages.

### 3.2. Effect of Tilt Angle

#### 3.2.1. Velocity Distribution

Velocity maps can effectively demonstrate variations in velocities. Velocity magnitude information is obtained on the coaxial twin-rotor profile using post-processing techniques, and the magnitude of the induced velocity can be inferred from the velocity superposition. Generally, in terms of the induced velocity, the greater the induced velocity, the less the vehicle is disturbed by the external environment and the weaker the aerodynamic interference of the upper and lower paddles. Therefore, a more comprehensive understanding of the aerodynamic characteristics of the coaxial twin-rotor system can be achieved by conducting an in-depth study of the velocity maps and induced velocities, thus providing an important reference for performance optimization and design improvement.

**Figure 12:**
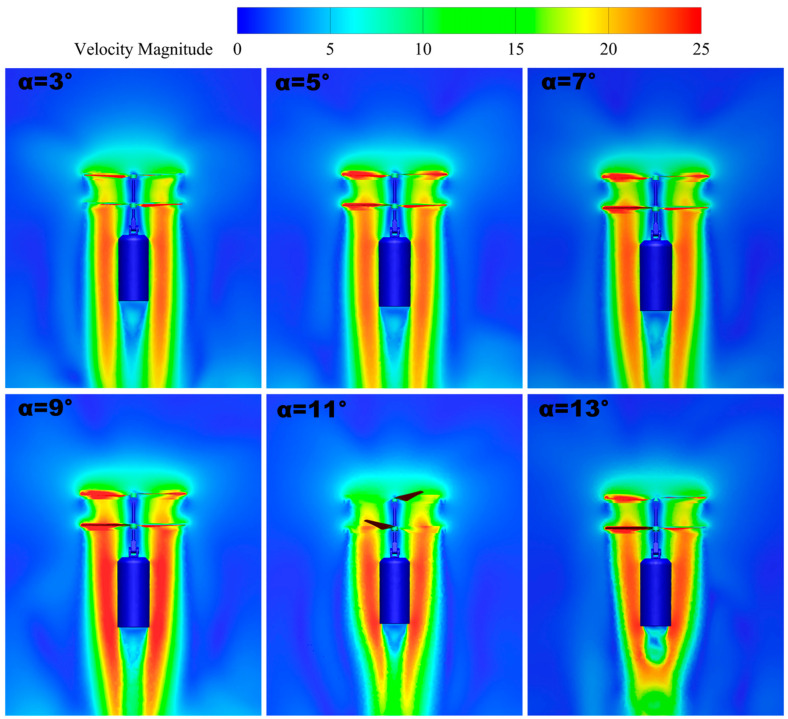
Velocity maps.

Figure 12 shows the velocity maps at a speed of 7000 RPM. From the velocity distribution in the graph, it can be seen that the coaxial twin-rotor UAV shows an increasing trend of velocity at the paddles as the tilt angle increases. When the tilt angle *δ* = 3° increases to *δ* = 9°, the velocity distribution of the wingtip is the largest. At a tilt angle of *δ* = 9°, the velocity is 77.9 m/s, which is a 15% increase compared with that at *δ* = 3°. When the tilt angle increases from *δ* = 9° to *δ* = 13°, the velocity distribution at the blade shows a decreasing trend. A tilt angle of *δ* = 9° results in a 35% increase in wingtip velocity compared with *δ* = 13°. By observing the velocity slice map of the coaxial twin-rotor UAV at the paddles, it can be seen that the red part of the area increases to the peak state when the tilt angle increases to *δ* = 9°, at which time the symmetry of the UAV’s velocity map is the best and the velocity distribution of the rotor is the largest.

**Figure 13:**
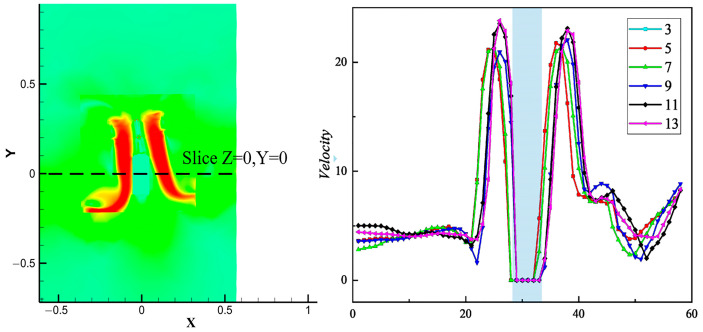
Slicing Z = 0, Y = 0 velocity profile.

Figure 13 shows the trend of velocity change in the slices Z = 0 and Y = 0. The locations labeled in the figure are the intervals where the fuselage loads are located. With an increase in the tilt angle, the lower induced velocity shifts to the opposite direction of the rotor tilt, and the rotor-induced velocity also shows a downward trend, which is favorable for the UAV to carry out attitude transformation. However, too large a tilt angle will lead to a large loss of UAV blade speed and poor stability, so this factor should be taken into account in the subsequent design.

#### 3.2.2. Vorticity Distribution

**Figure 14:**
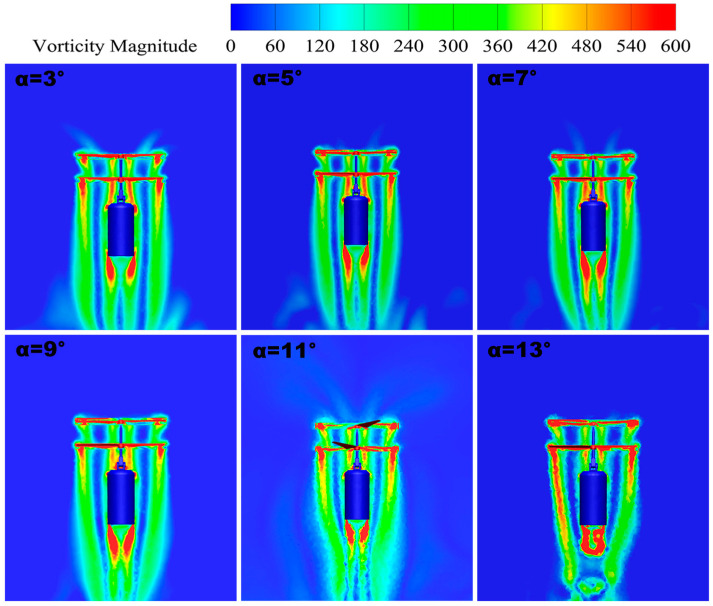
Vorticity maps.

The vorticity distribution of the UAV at tilt angles from *δ* = 3° to *δ* = 13° is shown in Figure 14. By observing the vorticity map in the figure, we can determine the pattern of airflow dynamics with the change in the tilt angle; when the tilt angle is too large or too small, the degree of disturbance between the upper and lower rotors significantly increases. The occurrence of this phenomenon may stem from the fact that a change in the tilt angle affects the diffusion of vortices in the rotor tip, leading to the mutual interference effect of the airflow in the vicinity of the rotor. This mutual interference will lead to the weakening of the rotor lift and will further weaken the stability of the airflow dynamics due to the vortex-induced velocity. Therefore, the influence of the change in the tilt angle on the aerodynamic performance is crucial, and its effect needs to be fully considered in the design and optimization process.

By conducting a more in-depth analysis of the vorticity maps, we find that the stability of the airflow is better at specific tilt angles. At a tilt angle of *δ* = 9°, the vortex flow is smoother, uniformly distributed, and organized, with weak interference from the double-rotor tip vortices and better overall symmetry. Therefore, this particular tilt angle provides better airflow stability.

#### 3.2.3. Turbulent Dissipation Rate

The turbulent dissipation rate is an important parameter when evaluating the aerodynamic characteristics of a coaxial biplane UAV. The turbulent dissipation rate is the rate at which turbulent energy is converted into thermal energy per unit of time, thus quantifying the decay of turbulent kinetic energy. The turbulent dissipation rate has a direct impact on the UAV’s lift generation and aerodynamic stability. A high turbulent dissipation rate means that the turbulence interference during flight is reduced more effectively, which, in turn, reduces flight resistance. Therefore, reasonable control of the turbulent dissipation rate can not only improve the flight performance of the UAV but can also enhance its aerodynamic stability so that it can perform better under various flight conditions.

**Figure 15:**
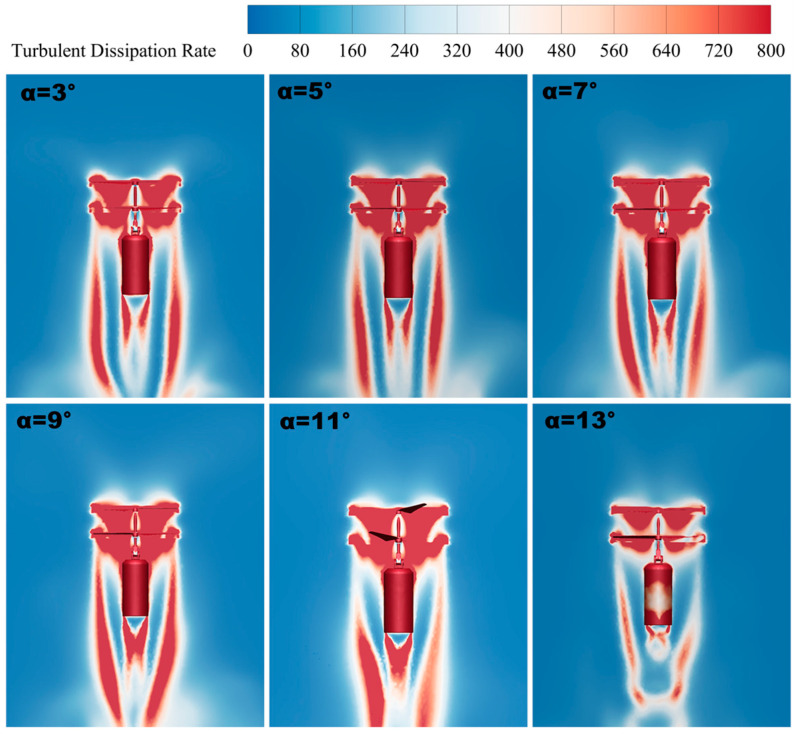
Turbulent dissipation rate slices.

When the tilt angle of a UAV changes, its aerodynamic profile and the distribution of forces in the wind field also change. This change may result in a change in the flow pattern of the surrounding airflow, which affects the strength of the turbulence and the dissipation rate. In Figure 15, it can be seen that, as the forward tilt angle increases, the turbulent dissipation rate shows an increasing trend at tilt angles from *δ* = 3° to *δ* = 9° (the larger the area of the red region in the figure, the higher the turbulent dissipation rate), and it shows a decreasing trend when the tilt angle of *δ* = 9° continues to increase to *δ* = 13°. Thus, the turbulent dissipation rate peaks at the tilt angle of *δ* = 9°. It can be seen that a higher turbulent dissipation rate helps to reduce the turbulent motion perturbation on the lift and improve the lift generation efficiency of the UAV, and its aerodynamic characteristics are also better. By observing the sliced plot of the turbulent dissipation rate, it is concluded that, when the coaxial twin-rotor UAV is tilted at *δ* = 9° with a higher turbulent dissipation rate, the rotor layout is more efficient, and it has better aerodynamic characteristics.

### 3.3. Effect of Load Thrust

**Figure 16:**
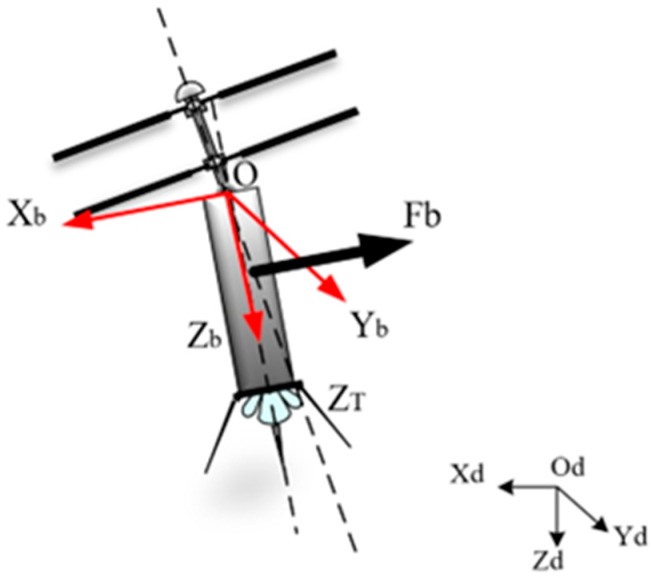
Definition of load thrust.

After vertical takeoff, the coaxial twin-rotor UAV needs to undergo an attitude transition from ascent to level flight, in which the twin-rotor provides both upward lift and horizontal forward force by tilting at a certain angle. Due to the relative motion relationship between the load below the rotor and the rotor, the wind resistance of the load below increases with the increase in the forward horizontal force. Additionally, when the wind resistance increases to a certain extent, it will push the load to rotate around the center of mass, and, at the same time, it will reduce the area of the wind resistance in the horizontal direction, which is conducive to the ascent-to-horizontal attitude adjustment of the coaxial twin-rotor UAV. This process is referred to as the rigid motion of the load around the center of mass, and the load thrust $F_{b}$, which is based on the loaded coordinate system $O-X_{b}Y_{b}Z_{b}$, is also an important parameter in the attitude transition process. The definition of the load thrust is shown in Figure 16 below.

In the initial state of the transition mode of a coaxial twin-rotor UAV, its rotor structure is tilted, while its fixed-wings do not generate forces at this time. The force and lift in the forward direction of the UAV are generated by the tilting rotor structure to provide it with different tilt angles horizontally and different thrusts on the load underneath the drone. According to the kinetic equations in the attitude conversion process, it can be seen that the important parameters affecting the attitude adjustment process are the aerodynamic velocity and angular velocity of the rotor. The rotor tilt angle affects the aerodynamic angular velocity, and the load thrust of the load below the UAV is also proportional to the aerodynamic velocity. Therefore, an aerodynamic simulation is used to study the magnitude of the load thrust underneath the UAV when its initial tilt angle is different.

**Figure 17:**
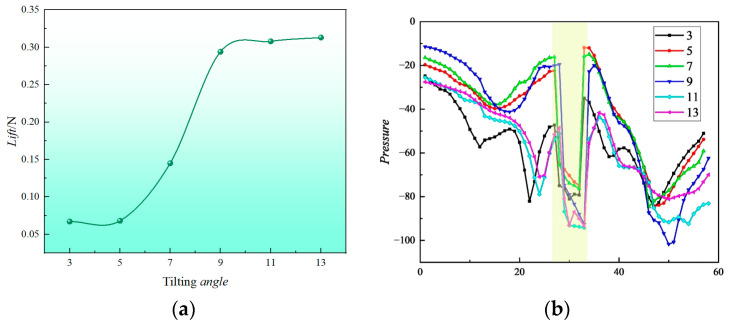
Load thrust and pressure curve. (**a**) Load thrust curve. (**b**) Slice Z = 0, Y = 0 pressure curve.

From an analysis of Figure 17a, it can be seen that, when the initial tilt angle *δ* = 3° is increased to *δ* = 9°, the thrust force on the load shows an increasing trend. The thrust force on the load increases significantly at a tilt angle of *δ* = 9°, which is 439% higher than that at *δ* = 3°. As the tilt angle continues to increase to *δ* = 13°, the trend of an increasing thrust force on the load slows down. Figure 17b displays a plot of sliced Z = 0 and Y = 0 pressure profiles, consistent with the slicing method shown in Figure 13. The positions labeled in the figure are the intervals where the fuselage loads are located. When the UAV is tilted, the pressure difference between the front and back of the load below is affected and changes accordingly, and the pressure change at the slice shows a nonlinear trend with the change in the tilt angle *δ*. The pressure difference between the front and back of the load changes the most when *δ* = 9°, which corresponds to the trend of the significant increase in the thrust of the load.

## 4. Conclusions

This study analyzes a model of tilt-powered coaxial twin-rotor UAVs and improves the theoretical model. The rotor layout is optimized through CFD calculations, and the influence of the upper and lower rotor spacing on the overall lift is examined. The influence of the change in the initial tilt angle on the load thrust and aerodynamic parameters is further investigated. The main results are as follows:

(1) Different rotor spacings of a UAV will affect its overall aerodynamic performance, and too large or too small a spacing is not conducive to the stabilization of its aerodynamic performance. When the rotor spacing ratio is h/R = 0.5, the rotor lift and the overall aerodynamic performance have obvious advantages. The optimum rotor spacing is determined to be h = 0.5 R.
(2) In the initial state, when performing the attitude adjustment process, the load thrust increases significantly when the initial tilt angle is *δ* = 9, being 439% higher than that at a tilt angle of *δ* = 3°. A larger load thrust facilitates the transition mode attitude adjustment and changeover.
(3) When the initial tilt angle is *δ* = 9°, the wing tip velocity reaches the maximum value of 77.9 m/s, which is 15% and 35% higher than that at *δ* = 3 and *δ* = 13°, respectively. In addition, the vortex flow is smoother and more uniformly and neatly distributed, and the disturbance of the twin-rotor wingtip vortex is relatively weak; meanwhile, with a high turbulent dissipation rate, the rotor layout is also more efficient.
(4) By considering the influence of the change in the initial tilt angle on the load thrust and aerodynamic parameters, it is found that, when the initial tilt angle is *δ* = 9, the aerodynamic layout is reasonable, the load thrust is larger, and the aerodynamic characteristics are optimized. The optimal initial tilt angle is determined to be *δ* = 9°. This conclusion is of guiding significance for the design and optimization of UAVs, aiding in improving their flight stability, maneuverability, and efficiency, and provides strong support for the development and application of UAV technology.

This study primarily relies on CFD simulations, with limited experimental validation. Future work will incorporate wind tunnel experiments and actual flight tests to further calibrate and validate the model. Additionally, the rotor layout optimization in this research is based on a specific UAV design; future studies will consider adaptability to various UAV structures and flight conditions. Lastly, we also plan to conduct long-term flight stability analyses in more complex flight scenarios to enhance the practical performance of UAVs.

## Data Availability

The original contributions presented in the study are included in the article; further inquiries can be directed to the corresponding author.
